# Cognitive Training Using Fully Immersive, Enriched Environment Virtual Reality for Patients With Mild Cognitive Impairment and Mild Dementia: Feasibility and Usability Study

**DOI:** 10.2196/18127

**Published:** 2020-10-14

**Authors:** Seo Jung Yun, Min-Gu Kang, Dongseok Yang, Younggeun Choi, Heejae Kim, Byung-Mo Oh, Han Gil Seo

**Affiliations:** 1 Department of Rehabilitation Medicine Seoul National University Hospital Seoul Republic of Korea; 2 Department of Computer Engineering Dankook University Gyeonggi Republic of Korea

**Keywords:** mild cognitive impairment, dementia, virtual reality, enriched environment

## Abstract

**Background:**

Cognitive training using virtual reality (VR) may result in motivational and playful training for patients with mild cognitive impairment and mild dementia. Fully immersive VR sets patients free from external interference and thus encourages patients with cognitive impairment to maintain selective attention. The enriched environment, which refers to a rich and stimulating environment, has a positive effect on cognitive function and mood.

**Objective:**

The aim of this study was to investigate the feasibility and usability of cognitive training using fully immersive VR programs in enriched environments with physiatrists, occupational therapists (OTs), and patients with mild cognitive impairment and mild dementia.

**Methods:**

The VR interface system consisted of a commercialized head-mounted display and a custom-made hand motion tracking module. We developed the virtual harvest and cook programs in enriched environments representing rural scenery. Physiatrists, OTs, and patients with mild cognitive impairment and mild dementia received 30 minutes of VR training to evaluate the feasibility and usability of the test for cognitive training. At the end of the test, the usability and feasibility were assessed by a self-report questionnaire based on a 7-point Likert-type scale. Response time and finger tapping were measured in patients before and after the test.

**Results:**

Participants included 10 physiatrists, 6 OTs, and 11 patients with mild cognitive impairment and mild dementia. The mean scores for overall satisfaction with the program were 5.75 (SD 1.00) for rehabilitation specialists and 5.64 (SD 1.43) for patients. The response time of the dominant hand in patients decreased after the single session of cognitive training using VR, but this was not statistically significant (*P*=.25). There was no significant change in finger tapping in either the right or left hand (*P*=.48 and *P*=.42, respectively). None of the participants reported headaches, dizziness, or any other motion sickness after the test.

**Conclusions:**

A fully immersive VR cognitive training program may be feasible and usable in patients with mild cognitive impairment and mild dementia based on the positive satisfaction and willingness to use the program reported by physiatrists, OTs, and patients. Although not statistically significant, decreased response time without a change in finger tapping rate may reflect a temporary increase in attention after the test. Additional clinical trials are needed to investigate the effect on cognitive function, mood, and physical outcomes.

## Introduction

Dementia, a major global public health burden, is a neurodegenerative disorder that impairs cognitive functions such as memory, language, and goal-directed behaviors [1]. Cognitive training in early stages of dementia has been considered a promising tool for addressing the impact of cognitive changes [2]. A recent systematic review demonstrated that computerized cognitive training improves cognition and psychosocial function in patients with mild cognitive impairment; however, there is lack of evidence in those with dementia [3]. Patients with cognitive impairment are less likely to engage and less able to concentrate in repetitive training compared with the healthy elderly population [4]. On the other hand, cognitive training based on virtual reality (VR) introduces motivational and playful aspects of training for patients with mild cognitive impairment and dementia [5].

Emerging VR cognitive training focuses on orientation, spatial navigation, face recognition, memory function, and instrumental activities of daily living [6]. The advantage of applying VR is that activities, tasks, and evaluation can occur in a secure environment [7]. Furthermore, VR training parameters can be adjusted within a person-centered therapeutic milieu [8]. A multisensorial experience through VR enables feedback-based learning [9].

Although virtual environments are artificial in nature, the user can elicit a rich feeling of realness and agency [10]. The immersion level of the VR system has a critical role on visual realism. The sense of “presence” refers to being and therefore how well the virtual environment represents the real world [11]. Reality level refers to the level at which a user truly experiences immersion [12]. Fully immersive VR sets people free from external interference. This VR world encourages patients with cognitive impairment to maintain selective attention [13]. However, VR technologies with sufficient levels of immersion or interaction have not been developed for this patient population [6].

Familiar image-based virtual environments can stimulate the recollections of autobiographical memory in healthy elderly [14]. Enriched environments provide conditions that enhance sensory, cognitive, and motor stimulation [15]. Furthermore, experiencing rich environments evokes positive emotions. A positive feeling is a protective factor against brain dysfunction, similar to that of the cognitive enrichment hypothesis [16]. It has been reported that enriched environments improve spatial impairment and memory deficits in vascular dementia and Alzheimer disease mouse models [17,18]. Recently, a video game that applied enriched environments was found to have a positive effect on cognitive function and mood in subjects with normal aging and mild to moderate cognitive impairment [4].

Only a few studies on cognitive training using fully immersive VR for patients with mild cognitive impairment and dementia have been conducted [13,19]. VR games based on enriched environments can be helpful for full immersion and comfort of patients experiencing cognitive decline. Furthermore, as it targets the elderly with cognitive impairment, it is necessary to develop VR games that reflect cultural specificity. We developed a fully immersive VR system based on enriched environments in order to train attention, memory, and executive function in the elderly. The purpose of this study was to investigate the feasibility and usability of cognitive training using the VR system with rehabilitation specialists and patients with mild cognitive impairment and mild dementia.

## Methods

### Study Design

This was a pilot study to evaluate the usability and feasibility of a fully immersive VR cognitive training program with an enriched environment. The study consisted of a single session and a survey completed by physiatrists and occupational therapists (OTs) as well as patients with mild cognitive impairment and mild dementia. This study protocol was approved by the Institutional Review Board of Seoul National University Hospital (IRB No. 1809-126-975) on October 16, 2018. All participants provided written informed consent. The study was performed under the principles of Good Clinical Practice and the Helsinki Declaration.

### VR Interface System

The VR interface system consisted of an HTC Vive head-mounted display and a custom-made hand motion tracking module developed for hand pose estimation and 3-dimensional positions of the hands in the working space.

**Figure 1:**
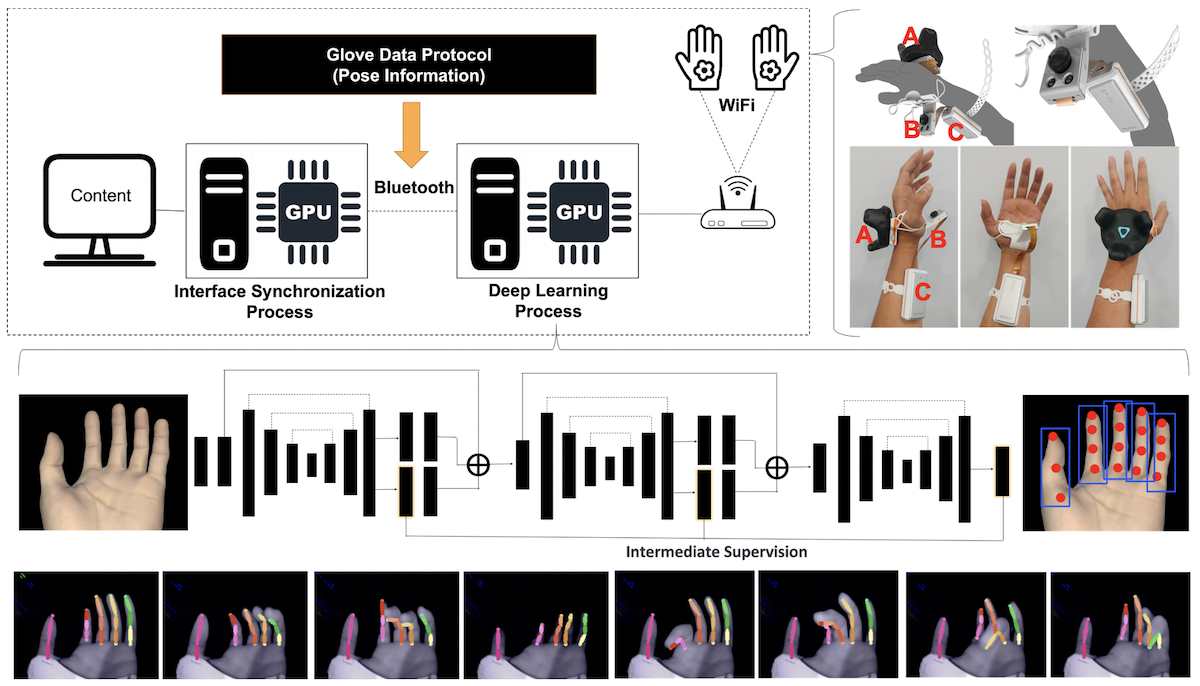
The overall system architecture of the module for hand pose estimation: (A) motion tracking module, (B) camera module, (C) transmission/reception module and battery.

Figure 1 shows the overall system architecture of the module for hand pose estimation in a virtual environment. The hand motion tracking module has a camera on the palm side of the hand to capture images; the camera is synced with a computer system for the deep learning process in order to estimate the hand poses. Each of the 2 modules for both hands transmits real-time hand images to the deep learning process in the computer system through WiFi communication. The deep learning process computes finger joint positions from the images to estimate hand poses in real time, which is delivered to the interface synchronization process in the computer system for the VR game through Bluetooth. HTC Vive trackers are installed on the backside of the hands to obtain the hands’ positions, which are also used to control the VR game.

The network model of our deep learning process was designed based on the hourglass network [20]. The hourglass network has primarily been used for human body pose estimation. Therefore, the structure of the network is not optimized for hand pose estimation. We mainly focused on the modification of the feature extraction layers of the network so that finger features could be efficiently extracted. However, as it was difficult to obtain sufficient hand image data for training, we used the composite hand data generator to generate simulated hand image data [21]. More than 2 million images were generated to train our deep learning process. As shown at the bottom of Figure 1, the 2-dimensional joint position of each finger was successfully extracted using our proposed method.

With the joint position data, we could estimate the finger joint angles from an inverse kinematics model. Moreover, without the inverse kinematics model, gestures like grip could be easily recognized from the joint position data. For example, grip could be detected if the vertical positions of fingertips were lower than the threshold value. Thumb-to-finger tap gesture could also be easily detected using only the 2-dimensional joint position data [22]. These data were then used to interface with the VR game.

The hand motion tracking module was designed such that patients could wear it easily without much effort. The silicon pad and strap made the module easy to wear, remove, and clean.

### VR Cognitive Training Program Based on an Enriched Environment

We developed virtual harvest and cook games in enriched environments representing Korean rural scenery. In all the games, the patients interacted with the environment from an egocentric point of view (known as “first person point of view”). The developed algorithm is shown in Multimedia Appendix 1.

**Figure 2:**
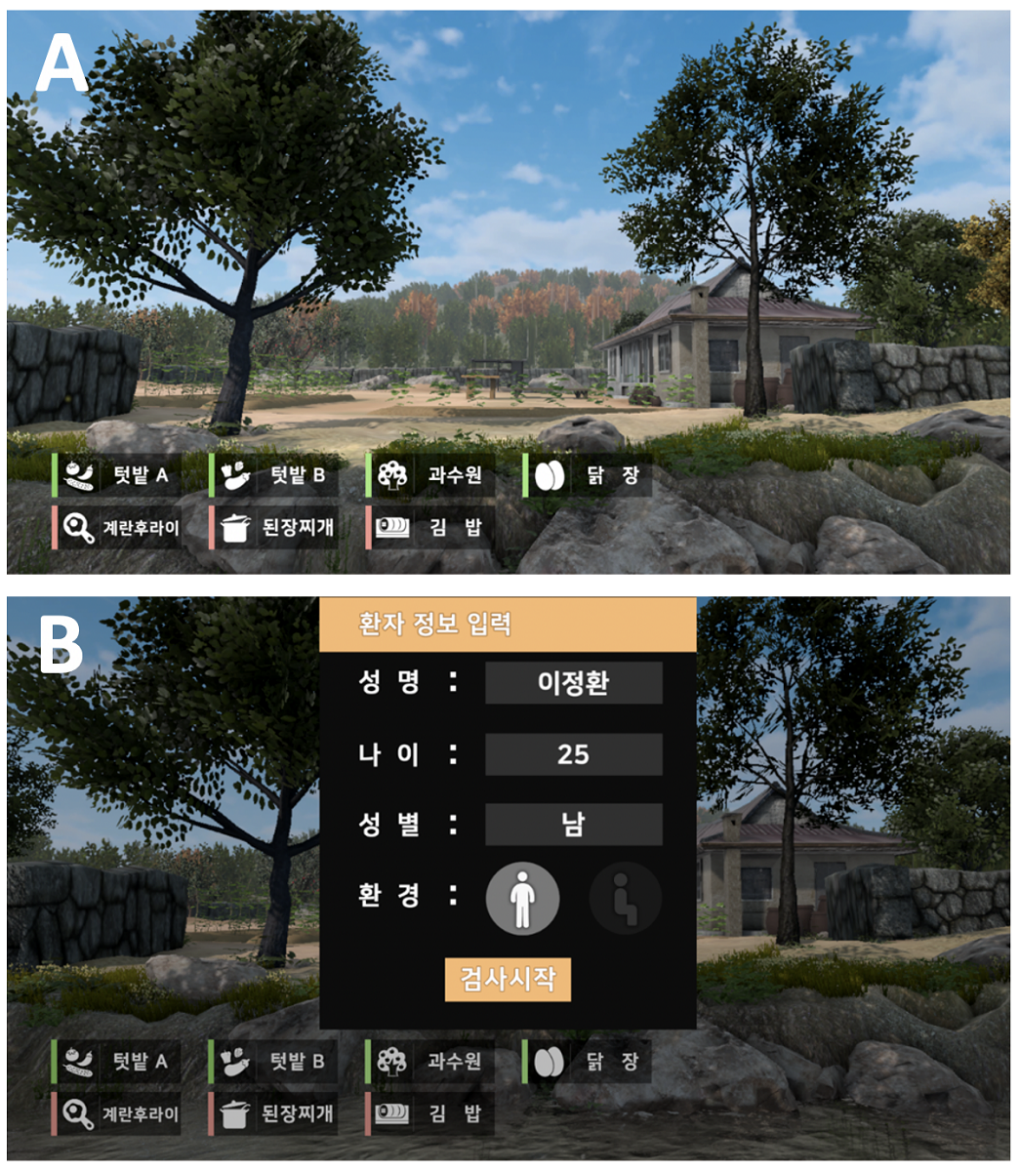
Default scenes in the program: (A) primary scene showing a usual Korean farm village and country house where one could harvest and cook and (B) patient information input scene.

In the default scene of the program, the user was standing in a stream with a view of a country field and house (Figure 2). Sounds of water, wind, birds, and soft music were played as background music. The scene mimicked an environment familiar and comfortable for Korean elderly patients.

**Figure 3:**
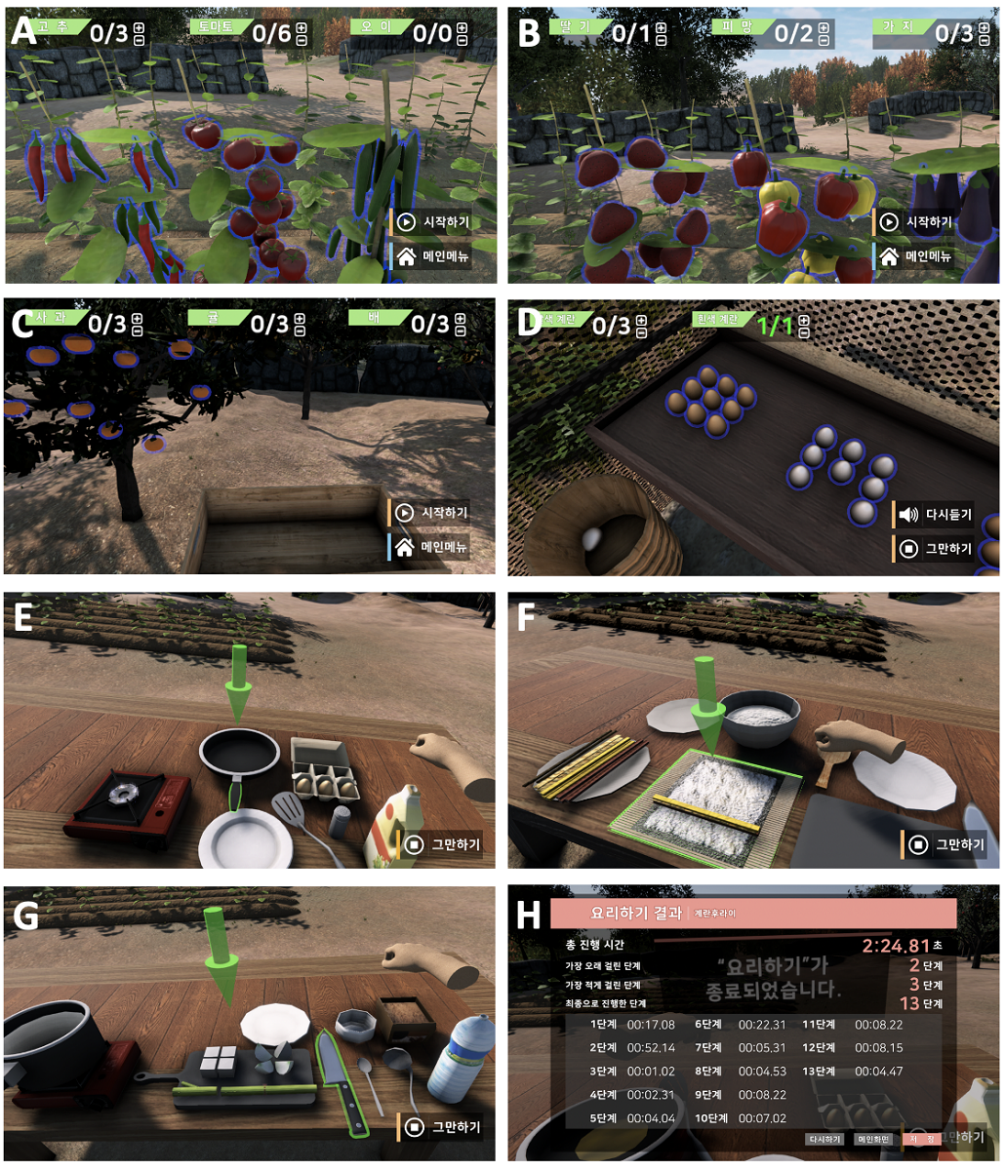
Harvest and cook game: (A) farm A (chilis, tomatoes, and cucumbers), (B) farm B (strawberries, paprika, and eggplants), (C) orchard (apples, mandarins, and pears), (D) hen house (brown eggs and white eggs), (E) fried eggs, (F) gimbap (dried seaweed roll), (G) soybean paste stew, (H) results of the cooking game.

The harvest game aimed to improve sustained, alternating, and selective attention and working memory (Figure 3). In the game, there were 4 backgrounds: farm A (chilis, tomatoes, and cucumbers), farm B (strawberries, paprika, and eggplants), an orchard (apples, mandarins, and pears), and a hen house (brown eggs and white eggs). The type and number of crops and eggs harvested and the time limit were controlled autonomously by the operator, depending on each participant’s performance level. After the operator inputted the harvest target and time frame, an artificial voice informed the patient. If the participants were unable to complete the task in the appointed time, visual cues with green lights around the object were provided. If the participant performed incorrectly, there was no limit but it remained on record. After the participants finished the task, the instructor checked time taken by the participant to complete the task, the number of times the direction was replayed, and the harvest type and number.

The cook game focused on improvements in sustained and selective attention; working, spatial, and procedure memory; and executive function. The cook program included 3 types of recipes: fried eggs, gimbap (dried seaweed roll), and soybean paste stew. The fried egg recipe consisted of 12 steps, gimbap recipe consisted of 11 steps, and soybean paste stew recipe consisted of 14 steps. The recipe was selected by the operator depending on the participant’s level. The participants were verbally instructed by the program on how to execute the allocated recipe. If a participant could not cook the recipe properly, a green arrow around the item necessary for the present step glowed. The strategies of the cook game were based on “errorless learning” [23]. If patients could not progress to the next step, the program did not progress to the next step. After the participants performed the task, the execution time was tabulated for each step.

The VR cognitive training program was evaluated and improved through the initial usability test by 12 physiatrists and 7 OTs before the final application.

### Procedure

Participants including physiatrists, OTs, and patients were provided with a description of the program objectives and procedures before the test. All participants were seated on a fixed chair, wearing hand motion tracking modules, and supervised by an OT during the test (Multimedia Appendix 2). The therapist was trained to manipulate the software for the usability test and to deal with adverse events. The OT adjusted game difficulty to match the patient’s level based on the Mini-Mental State Examination (MMSE) score and Clinical Dementia Rating (CDR). Physical and verbal assistance was provided for participants who struggled to understand the instructions properly. The participants experienced all 4 harvest games and 3 cooking games. The usability and feasibility test was a single session lasting of 30 minutes.

### Participants

Physiatrists and OTs with more than 1 year of clinical experience were recruited to test the usability and feasibility of the developed program. Patients were recruited from a tertiary hospital. A total of 15 patients were screened for eligibility for study inclusion. Inclusion criteria were age ≥65 years and a score of more than 19 and less than 28 points on the MMSE. Exclusion criteria were patients with moderate to severe dementia who could not understand the research content or agree voluntarily, a history of severe dizziness or epilepsy, psychiatric symptoms or behavioral problems that made it difficult to participate in the study, and other severe medical problems such as neurological or orthopedic diseases.

### Outcome Measures

Physiatrists and OTs evaluated the usability of the developed VR cognitive training system using a self-report questionnaire based on a 7-point Likert-type scale. Questionnaire items consisted of 6 categories and 22 items: overall satisfaction, acceptability (needs, favorability, effectualness for mild cognitive impairment and mild dementia, and distinction), satisfactoriness (intent adequacy, setting adequacy, setting immediacy, inducement of interest, and content diversity), expectation effectiveness (attraction of voluntary participation from patients, labor reduction for therapists, and contribution to therapists’ work), stability (confirmation of proper equipment positioning, warning of system errors, external appearance of safety, durability, and stability satisfaction), and others (willingness to use, additional use intention with other therapeutic tools, and recommendation intention). The higher the score, the more positive the result. The survey also included additional suggestions regarding the developed system.

The patients’ baseline characteristics, including age, sex, medical history, MMSE, CDR, and Geriatric Depression Scale (GDS), were assessed. At the end of the experience, the feasibility of the enriched environment VR games was assessed by patients using a self-report questionnaire with a 7-point Likert-type scale based on 9 items (overall satisfaction, interest, mood, motivation, difficulty, discomfort, anxiety, willingness to use, and expectations for VR rehabilitation). The survey also included additional descriptions of subjective experiences.

Reaction time was assessed to evaluate the activity process of the central nervous system, such as motor preparation and motor program [24]. Using an iPad app (Reaction Test & Speed Test, Wang Haiwen), the instructor instructed patients that when the red screen became green, the screen should be touched as quickly as possible. This app measured response time from the color change to the screen touch. Patients tried for a total of 5 times using their dominant hand.

To assess motor speed, the finger tapping test was used [25]. Participants tapped just an iPad (digital finger tapping test, SyBu Data) with the index finger as fast as they could for 10 seconds. An average of 3 trials with both hands was completed.

### Statistical Analysis

Baseline characteristics and assessment data are expressed as mean and SD for continuous variables. The Wilcoxon signed-ranks test was used to evaluate changes in results before and after the usability test. The Mann-Whitney test was employed to compare the number of finger taps between the right and left hands. A *P* value <.05 was considered statistically significant. SPSS Statistics 21.0 for Windows (IBM Corp, Armonk, NY) was used for all analyses.

## Results

In this study, 10 physiatrists and 6 OTs (mean experience: 4.9 years, range 2-30 years) participated on March 21, 2019. The overall satisfaction score of the system was 5.75 (SD 1.00). In descending order, the mean score was highest for acceptability (5.79, SD 0.16), expectation effectiveness (5.32, SD 0.43), satisfactoriness (5.11, SD 0.79), and stability (4.86. SD 0.32; Table 1).

A total of 11 patients with mild cognitive impairment and mild dementia were enrolled in this study between April 26, 2019 and May 17, 2019. The average age of the participants was 72.64 years (SD 4.65 years; Table 2). The MMSE score ranged from 23 to 28 points, and the mean MMSE score was 26.91 points (SD 1.58 points). The CDR was 0.5 in 8 patients and stage 1 in 3 patients. The mean baseline GDS score was 17.55 points (SD 6.30 points, range 10-30 points). All patients were right-handed.

**Table 1:** Physiatrists’ and occupational therapists’ self-report questionnaire results on a 7-point Likert-type scale. The higher the score, the more positive the result.

Scale and subscale		Score, mean (SD)	
Overall satisfaction		5.75 (1.00)	
**Acceptability**		5.79 (0.16)	
	Needs		5.88 (0.89)
	Favorability		6.00 (0.82)
	Effectualness (mild cognitive impairment)		5.63 (0.89)
	Effectualness (mild dementia)		5.63 (0.81)
	Distinction		5.81(1.17)
**Satisfactoriness**		5.11 (0.79)	
	Intent adequacy		5.38 (0.89)
	Setting adequacy		4.19 (1.33)
	Setting immediacy		4.50 (1.37)
	Inducement of interest		6.19 (0.75)
	Content diversity		5.31 (1.54)
**Expectation effectiveness**		5.32 (0.43)	
	Attraction of voluntary participation from participants		5.75 (1.06)
	Labor reduction for therapists		4.89 (1.61)
	Contribution to therapists’ work		5.31 (0.95)
**Stability**		4.86 (0.32)	
	Confirmation of proper equipment positioning		5.00 (1.46)
	Warning of system errors		4.50 (1.60)
	External appearance of safety		5.00 (1.10)
	Durability		4.56 (1.26)
	Stability satisfaction		5.25 (1.24)
**Others**		5.79 (0.14)	
	Willingness to use		5.63 (1.41)
	Additional use intention with other therapeutic tools		5.88 (0.89)
	Recommendation intention		5.88 (0.81)

**Table 2:** Patients’ demographics and baseline characteristics (n=11).

Number	Age (years)	Sex	MMSE^a^ (points)	CDR^b^ (points)	GDS^c^ (points)
1	72	M^d^	28	0.5	22
2	74	F^e^	28	0.5	30
3	71	F	27	1	21
4	76	M	27	0.5	14
5	81	M	25	0.5	16
6	76	M	28	1	24
7	67	F	28	0.5	10
8	76	F	28	0.5	15
9	73	M	23	1	10
10	67	F	27	0.5	12
11	66	F	27	0.5	19

^a^MMSE: Mini-Mental State Examination.

^b^CDR: Clinical Dementia Rating.

^c^GDS: Geriatric Depression Scale.

^d^M: male.

^e^F: female.

For overall satisfaction with the program, the mean patient rating was 5.64 (SD 1.43). The study items with high scores were comfort (6.91, SD 0.30), anxiety (6.27, SD 1.62), and mood (6.18, SD 1.40). The remainder of the scores were as follows: interest, 5.82 (SD 1.33); motivation, 5.36 (SD 1.57); difficulty, 5.45 (SD 1.51); willingness to use, 5.82 (SD 1.83); and expectations for VR rehabilitation, 5.55 (SD 1.44). The mean response time of the dominant hand decreased after a single session of cognitive training using VR, but the difference was not statistically significant (pre-VR: 612.18 ms, SD 186.35 ms; post-VR: 546.82 ms, SD 130.64 ms; *P*=.25). There was no change in finger taps of the right (pre-VR: 37.97, SD 11.93; post-VR: 38.59, SD 11.75; *P*=.48) or left hand (pre-VR: 35.81, SD 11.59; post-VR: 33.85, SD 10.82; *P*=.42). There was no significant difference in the mean number of finger taps between the right and left hands before (right: 37.97, SD 11.93; left: 35.81, SD 11.59; *P*=.56) and after (right: 38.59, SD 11.75; left: 33.85, SD 10.82; *P*=.30) the training.

None of the participants reported headaches, dizziness, or any other form of motion sickness after the test.

## Discussion

### Principal Findings

We developed a memory and attention training program using enriched VR environments for patients with mild cognitive impairment and mild dementia. Our results suggest that the VR cognitive training system is feasible and usable based on the feedback received from physiatrists, OTs, and patients with mild cognitive impairment and mild dementia. None of the participants complained of motion sickness, and no adverse events occurred. Although not statistically significant, the decreased response time without changing the rate of finger tapping in patients may reflect a temporary increase in attention after the test.

Fully immersive VR can induce psychological immersion and high presence. Lifelike VR may narrow the gap between reality and the virtual world [26]. The high level of immersion and visual realism trigger autobiographical memories [27]. A combination of the enriched environment, which is almost like reality, and VR may enable patients with cognitive decline to feel emotionally stable. Neuropsychiatric symptoms including depression, apathy, and agitation occur in most patients with dementia [28]. The patients in this study were found to be mildly depressive as per their GDS score. After the test, the patients gave positive feedback on the questionnaire with regards to comfort, anxiety, and mood. Therefore, the program might be helpful due to improvement in mood in patients with mild cognitive impairment and mild dementia. This finding is in line with a report stating psychosocial intervention through individualized reminiscence and multisensory stimulation evoked emotional and social benefits for those with dementia, along with a preserved sense of identity [29].

In this study, allowing free hand movements in elderly patients who are not used to the VR machine operation ensured that they could access the VR environment more comfortably. Physiatrists, OTs, and patients did not report incommodiousness of a custom-made hand module. However, 3 rehabilitation specialists suggested that it would be better to add haptic feedback when grasping objects and to enhance the sensitivity of the motion detector. To bring about interactions between the user and the virtual environment, several input devices are needed [30]. A potential problem with hand-held devices is that a stiff controller limits kinematic error and completes the actions without continuous participation from the patient, which may limit the effect of training. Furthermore, an unsolved problem regarding the motion tracking systems and instrumented gloves is that they are limited when creating accurate user movements in the virtual environment [31]. In addition, bacterial contamination and sterilization could be an issue for medical use of hand-held devices and instrumented gloves. There is an ongoing need to make intuitive input devices for the freedom to manipulate devices and increase accuracy. Although the hand motion tracking module we developed is a prototype, it could compensate for several shortcomings of existing products.

It is also necessary to consider the needs of caregiving staff when designing and developing interventional VR programs. Physiatrists and rehabilitation therapists have many training interface options to choose from, including VR, video gaming, and other tablet-based applications [32]. A survey of rehabilitation specialists showed high overall satisfaction and intent to use the VR system for further training. However, they reported that the hardware system needs to be improved with regards to setting the speed and adequacy. It is also worth considering incorporating a haptic device into the hardware system to make patients with cognitive impairment more comfortable with their VR tasks and to provide them the appropriate tactile stimuli. Because the hardware system is not commercialized, continuous development is needed to improve user convenience.

### Limitations

This study has several limitations. First, the short test time may be insufficient to influence cognitive components and motor function. Because the primary purpose of this study was to evaluate the usability and feasibility of the developed system, all participants underwent a single session of VR cognitive training. Response time decreased after the short-term VR experience in patients although it was not statistically significant. This result may suggest improved cognitive process without changes in motor speed after VR cognitive training, as no changes were noted in the finger tapping test. To evaluate the efficacy of the developed program, a further clinical trial should be designed including several training sessions and a conventional cognitive training control group. Second, motion sickness was not evaluated objectively in this study. In order to minimize motion sickness during the VR experience, the researchers observed the condition of participants carefully and continuously asked them about any inconvenience. Participants in this study did not complain of motion sickness. Nevertheless, cybersickness due to VR use must be considered, especially when fully immersive VR is being utilized in cognitively impaired patients [33]. An objective assessment that can measure motion sickness symptoms such as dizziness, nausea, and headache may be required in future studies. Last, the developed program was only used with those with mild cognitive impairment and mild dementia. This study population was chosen based on previous studies that have reported the effect of computerized cognitive training in people with cognitive impairment [3]. However, as the enriched VR environment may be beneficial for reminiscence, relaxation, and enjoyment for different stages of dementia, future studies are warranted in patients with moderate and severe dementia.

### Conclusions

We developed a fully immersive VR cognitive training program with an enriched environment for patients with mild cognitive impairment and mild dementia. The feasibility and usability of the program were verified based on the positive satisfaction and willingness to use reported by physiatrists, OTs, and patients. Although we were not able to identify functional changes with the use of a single session, there may be an increase in attention following training. Additional clinical trials are needed to confirm the effects of this program on cognitive function, mood, and physical outcomes.

## Appendix

Multimedia Appendix 1 Algorithm for program development.

Multimedia Appendix 2 A patient experiences the virtual reality training with the head mount display (blue circle) and hand motion tracking module (red circles).

## Abbreviations

CDR: Clinical Dementia Rating
F: female
GDS: Geriatric Depression Scale
M: male
MMSE: Mini-Mental State Examination
OT: occupational therapist
VR: virtual reality
