# Supplementary figures and images for: Cognitive Training Using Fully Immersive, Enriched Environment Virtual Reality for Patients With Mild Cognitive Impairment and Mild Dementia: Feasibility and Usability Study
Source: JMIR Serious Games. 2020 Oct 14;8(4):e18127. doi: 10.2196/18127 (PMC7593866; doi:10.2196/18127)

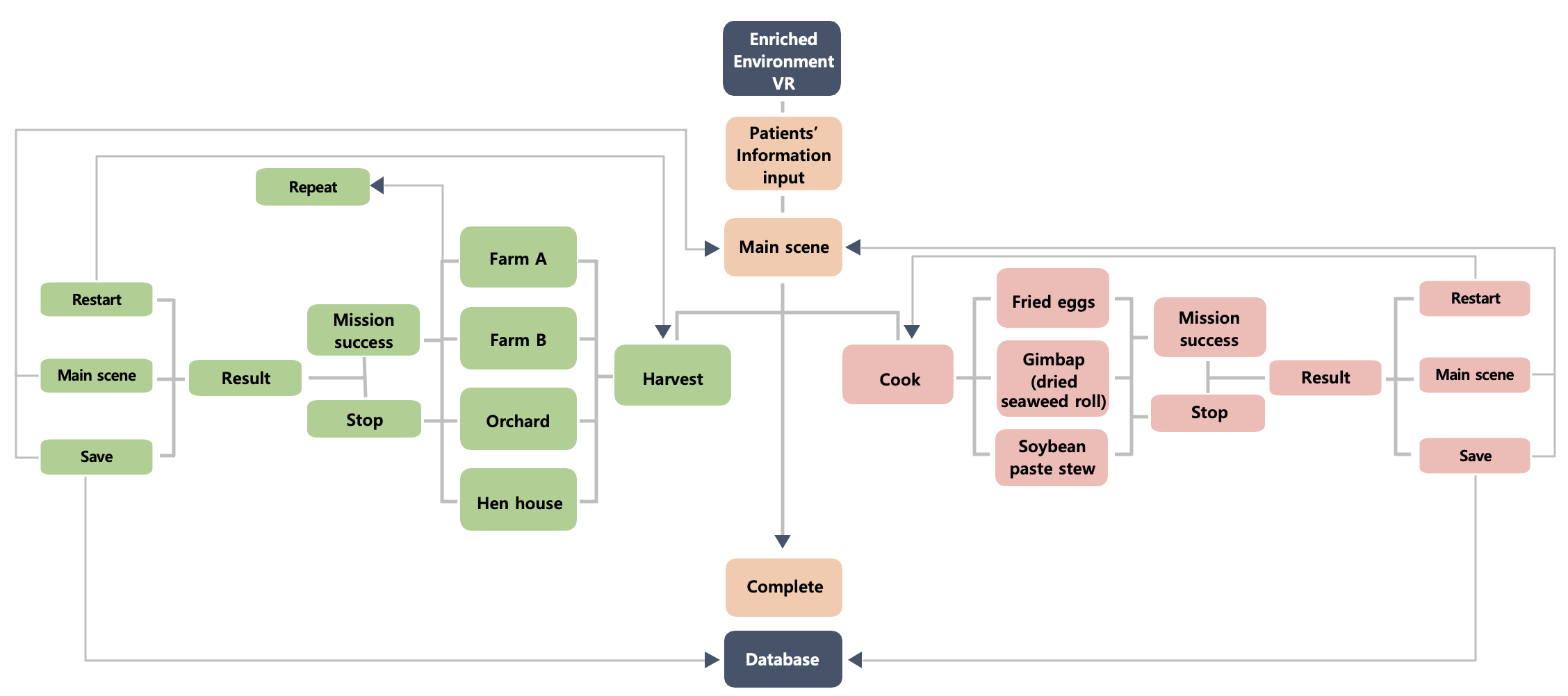

Supplement: Multimedia Appendix 1 [file games_v8i4e18127_app1.png]

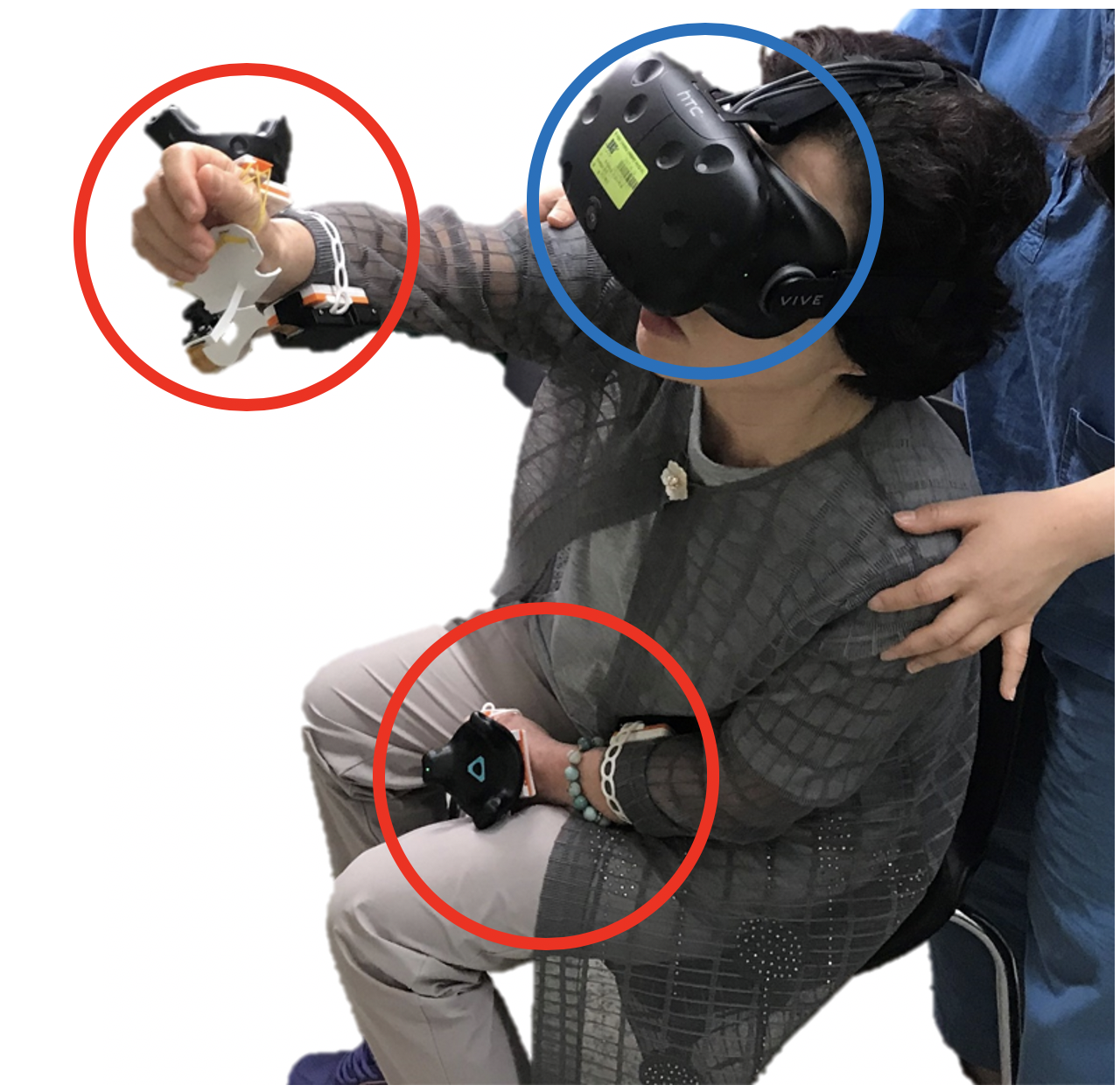

Supplement: Multimedia Appendix 2 [file games_v8i4e18127_app2.png]
